# 3-Mercaptopyruvate Sulfurtransferase, Not Cystathionine β-Synthase Nor Cystathionine γ-Lyase, Mediates Hypoxia-Induced Migration of Vascular Endothelial Cells

**DOI:** 10.3389/fphar.2017.00657

**Published:** 2017-09-20

**Authors:** Beibei Tao, Rui Wang, Chen Sun, Yichun Zhu

**Affiliations:** Shanghai Key Laboratory of Bioactive Small Molecules and Research Center on Aging and Medicine, Department of Physiology and Pathophysiology, Fudan University Shanghai Medical College Shanghai, China

**Keywords:** 3-mercaptopyruvate sulfurtransferase, cystathionine β-synthetase, cystathionine γ-lyase, hypoxia, hydrogen sulfide, migration, angiogenesis

## Abstract

Hypoxia-induced angiogenesis is a common phenomenon in many physiological and patho-physiological processes. However, the potential differential roles of three hydrogen sulfide producing systems cystathionine γ-lyase (CSE)/H_2_S, cystathionine β-synthase (CBS)/H_2_S, and 3-mercaptopyruvate sulfurtransferase (MPST)/H_2_S in hypoxia-induced angiogenesis are still unknown. We found that minor hypoxia (10% oxygen) significantly increased the migration of vascular endothelial cells while hypoxia (8% oxygen) significantly inhibited cell migration. The present study was performed using cells cultured in 10% oxygen. RNA interference was used to block the endogenous generation of hydrogen sulfide by CSE, CBS, or MPST in a vascular endothelial cell migration model in both normoxia and hypoxia. The results showed that CBS had a promoting effect on the migration of vascular endothelial cells cultured in both normoxic and hypoxic conditions. In contrast, CSE had an inhibitory effect on cell migration. MPST had a promoting effect on the migration of vascular endothelial cells cultured in hypoxia; however, it had no effect on the cells cultured in normoxia. Importantly, it was found that the hypoxia-induced increase in vascular endothelial cell migration was mediated by MPST, but not CSE or CBS. The western blot analyses showed that hypoxia significantly increased MPST protein levels, decreased CSE protein levels and did not change CBS levels, suggesting that these three hydrogen sulfide-producing systems respond differently to hypoxic conditions. Interestingly, MPST protein levels were elevated by hypoxia in a bi-phasic manner and MPST mRNA levels increased later than the first stage elevation of the protein levels, implying that the expression of MPST induced by hypoxia was also regulated at a post-transcriptional level. RNA pull-down assay showed that some candidate RNA binding proteins, such as nucleolin and Annexin A2, were dissociated from the 3′-UTR of MPST mRNA in hypoxia which implied their involvement in MPST mRNA regulation.

## Introduction

Angiogenesis, a process that mainly includes the proliferation, migration, and tube formation of vascular endothelial cells, is a common phenomenon in many physiological and patho-physiological processes, including organ development, wound healing, tumor development, and metastasis (Folkman and Shing, 1992; Folkman, 1995). New vasculature is usually formed when ischemia occurs, by sprouting from preexisting vessels, in order to maintain oxygen homeostasis (Folkman and Shing, 1992). Under chronic ischemic conditions, for example chronic myocardial ischemia, collateral circulation can be established to compensate for ischemia (Banai et al., 1994). The mechanism underlying hypoxia-induced angiogenesis usually involves an increase in hypoxia-inducible factors (HIF-1) and vascular endothelial growth factor (VEGF) regulated by HIF-1 (Semenza, 2000; Tang et al., 2004; Park et al., 2010).

Hydrogen sulfide can be endogenously produced by the hydrogen sulfide producing enzymes cystathionine γ-lyase (CSE), cystathionine β-synthetase (CBS), and 3-mercaptopyruvate sulfurtransferase (MPST) with L-cysteine and homocysteine as their substrates (Nagpure and Bian, 2016). CSE and CBS are pyridoxal-5′-phosphate (PLP) dependent, while MPST is not PLP dependent and uses 3-mercaptopyruvate as substrate, which is derived from cysteine in the presence of α-ketoglutarate. This reaction is catalyzed by the enzyme cysteine aminotransferase (Shibuya et al., 2009a). It has been reported that both exogenous and endogenous hydrogen sulfide has a proangiogenic effect. Exogenous H_2_S significantly increased vascular endothelial cell migration and *in Vitro* tube formation as assessed by counting tube length and branching point numbers and was proangiogenic in an *in Vivo* matrigel plug assay (Cai et al., 2007). Using CSE-knockout mice, Papapetropoulos et al. reported that endogenous H_2_S produced by CSE has a proangiogenic effect. They also reported that exogenous H_2_S increased vascular length in chicken chorioallantoic membranes (Papapetropoulos et al., 2009). Moreover, NaHS (H_2_S donor) significantly promoted collateral vessel growth and increased regional blood flow in ischemic tissue in a rat model of critical limb ischemia (Wang et al., 2010). In addition, the proangiogenic role of H_2_S has been reported in a mouse model of critical limb ischemia (Bir et al., 2012), in a chronic heart failure model of myocardial infarction (Qipshidze et al., 2012), and in a cerebral ischemia model (Jang et al., 2014).

We had previously reported that CSE and CBS are probably located differently in vascular endothelial cells. CSE is mainly localized near the membrane of vascular endothelial cells, while CBS appears to be distributed evenly in the cytoplasm (Tao et al., 2013). In addition, MPST has been reported as mainly localized in mitochondria (Nagahara et al., 1998; Shibuya et al., 2009b; Tanizawa, 2011). Therefore, we speculated that endogenous hydrogen sulfide produced by different systems, CSE/H_2_S, CBS/H_2_S, or MPST/H_2_S might have different target molecules since H_2_S could be easily oxidized and H_2_S levels could be reduced to an ineffective concentration if the target protein was far from the H_2_S producing enzyme.

Therefore, in the current study, the different roles of CSE/H_2_S, CBS/H_2_S, or MPST/H_2_S in mediating the effect of vascular endothelial cell migration induced by hypoxia were investigated, since vascular endothelial cell migration is essential to angiogenesis (Lamalice et al., 2007).

## Materials and methods

### Cell culture

Primary human umbilical vein endothelial cells (HUVECs) and culture medium (supplemented with or without 10% fetal bovine serum) were purchased from Allcells (Shanghai, China). Cells were cultured in gelatin-coated dishes at 37°C in an incubator containing 21% O_2_ for normoxia and 8 or 10% O_2_ for hypoxia, as well as 5% CO_2_. Cells were used for experiments within six passages.

### Wound healing assay

Confluent HUVECs were starved overnight and were scratched using a pipette tip for the wound healing assay. Markings were drawn on the culture dishes as reference points so as to make sure that the same visual field was photographed at 0 and 24 h. Typically, 8–12 visual fields were chosen randomly in one culture dish. Cells were photographed using an EVOS fl Microscope (Advanced Microscopy Group, Mill Creek, Washington) after incubation in normoxic or hypoxic conditions. The outline of the wound area (the area with no cells) was then drawn using Image J software to get the exact pixel coverage of each wound area. Then, the pixel coverage value of each wound area was converted to a standard value, shown as one data point, by being divided by a fixed value so as to normalize the average value of the control group to be 100.

### RNA interference

siRNAs specific for human CSE, CBS, or MPST are as follows: human CSE siRNA: sense 5′-CUAUGUAUUCUGCAACAAATT-3′, antisense 5′-UUUGUUGCAGAAUACAUAGAA-3′; human CBS siRNA: sense 5′-CUCACAUCCUAGACCAGUATT-3′, antisense 5′-UACUGGUCUAGGAUGUGAGAA-3′; human MPST siRNA: sense 5′-GGAAUUCCGUUGACUUGUUTT-3′, antisense 5′-AACAAGUCAACGGAAUUCCTG-3′.

siRNA specific for human CSE, CBS, MPST, and scrambled siRNA as a negative control were purchased from Thermo Scientific-Ambion. HUVECs were transfected using Lipofectamine RNAiMAX (Thermo Scientific-Invitrogen) and OPTI-MEM (Thermo Scientific-Gibco) according to the instructions provided by the manufacturer and then the cells were cultured for another 48 h for further analyses.

### Western blot analysis

Confluent HUVECs were starved overnight and were cultured in normoxic or hypoxic conditions. Then, cells were lysed in a 1 × sodium dodecyl sulfate (SDS) sample buffer (62.5 mM Tris-HCl pH 6.8, 10% glycerol, 2% w/v SDS). Bicinchoninic acid (BCA) reagent was purchased from Shen Neng Bo Cai Corp. (Shanghai, China) and the BCA method was used for protein concentration determination. Protein samples (50 μg) were subjected to electrophoresis in a 10% SDS-polyacrylamide gel, and then transferred to a polyvinyl difluoride membrane (Millipore-Upstate Biotechnology, Lake Placid, New York, USA). After blocking in 5% non-fat milk in Tris Buffered Saline with Tween-20 (TBST, 0.05% Tween-20) for 2 h at room temperature (RT), the membrane was incubated with primary antibodies at 4°C overnight. Anti-CBS and anti-β-actin were purchased from Proteintech Group (Chicago, IL, USA). Anti-CSE and anti-MPST were purchased from Santa Cruz Biotechnology (CA, USA). After washing the membranes using TBST and then incubating them with horseradish peroxidase-conjugated secondary antibodies for 2 h at RT, SuperSignal West Pico Chemiluminescent Substrate (Thermo-Pierce Biotechnology, Rockford, USA) was used for band detection. Band signals were then quantified using Smart viewer software.

### Real-time PCR

Total RNA was extracted from cells using Trizol reagent. RNA was reverse-transcribed using a cDNA synthesis kit (Toyobo Life Science, Japan). Real-Time PCR was performed using a StepOnePlus Real-Time PCR Detection System (Applied Biosystems Inc., CA, USA). A total volume of 20 μL reaction mixture containing 1 μL cDNA, 10 μL SYBR Green PCR Master Mix (Toyobo, Japan) and 1.6 μL each primer (10 μM) was used for this assay. GAPDH was used as reference for normalization and the relative expression of mRNA was calculated according to the ΔΔCt method.

The primer pairs used for the Real-time PCR were as follows: for the human MPST gene, the forward primer was 5′-CGGAGTCTCCTCCCTTTGGT-3′, and the reverse primer was 5′-CCTCCCTAAGATGCAGCTCG-3′. For the GAPDH gene, the forward primer was 5′-TGCCCCCATGTTCGTCA-3′, and the reverse primer was 5′-CTTGGCCAGGGGTGCTAA-3′.

### Plasmid constructs

The human MPST mRNA 5′-UTR and 3′-UTR were amplified from HUVECs using the following primers: 3′-UTR, the forward primer was 5′-AGCTGGGCAGGACACAGG-3′, and the reverse primer was 5′-TTCCTCAAAAATAAAACAGAAAGGAGG-3′. 5′-UTR-exon1, the forward primer was 5′-GGAAGGCGCGGGCAGCAGCGGCTCCGAGTGGCCGCGGCGGTGGGCTGTGCC-3′, the reverse primer was 5′-ATTGGCGACCTGCAGCGGACCAAAGGGAGGAGACTCCGGCACAGCCCACCGCC-3′. 5′-UTR-exon2, the forward primer was 5′-CGCTGCAGGTCGCCAATATAAATGCTTGATGAG-3′, the reverse primer was 5′-CTGCGGGCCTCCACTTGCTGGGCAGG-3′. 5′-UTR-exon3, the forward primer was 5′-AAGTGGAGGCCCGCAGCCCGAGTGTCGCCGCC-3′, the reverse primer was 5′-GGCGGCGACACTCGGGCTGCGGGCCTCCACTT-3′. The 3′-UTR or 5′-UTR (exon1, exon2, and exon3 jointed together) was subcloned into the pGEM-T vector.

### Generation of biotinylated 3′UTR and 5′UTR

RNA transcripts were produced by T7 RNA polymerase (Roche, Basel, Switzerland) with Biotin RNA labeling Mix (Roche, Basel, Switzerland) in an 18 μl system. After incubation for 2 h at 37°C, 2 μL RNase-free DNase was added and incubated for 15 min at 37°C to remove template DNA. Then, 2 μL 0.2 M EDTA (pH = 8.0) was added to stop the reaction. Biotinylated RNA was added into the Structure Buffer (10 mM Tris pH = 7.0, 0.1 M KCl, 10 mM MgCl_2_) to form the secondary structure.

### Biotin-RNA pull-down

The biotinylated RNA was bound to streptavidin-coated Dynabeads (Thermo Scientific-Invitrogen) according to the instructions provided by the manufacturer. Biotin-labeled antisense RNA was used as a control in all experiments. Magnetic beads were washed three times in 500 μL Wash Buffer I (10 mM Tris-Cl, pH 7.0, 1 mM EDTA, 2 mM NaCl) and resuspended in Wash Buffer I. Biotinylated RNA was incubated with magnetic beads at 4°C overnight. Biotinylated RNA-streptavidin magnetic beads were incubated with 1 mg cell extract with RNase inhibitor at RT for 1 h. The RNA/protein/bead complexes were washed three times using Wash Buffer II (10 mM Tris-Cl, pH 7.0, 1 mM EDTA, 100 mM KCl, 0.1% Triton X-100, 5% glycerol, 1 mM DTT). Then, proteins were eluted by boiling in 5 × SDS PAGE loading buffer and resolved using SDS-PAGE. Protein bands were silver-stained and cut for mass-spectrometric analysis (Lu Ming Corp. Guangzhou, China).

### Tryptic digestion and sample preparation for LC-collision-induced dissociation-MS-MS analysis

Protein bands from SDS-PAGE gels were excised. The gel fragments were cut into small pieces and decolored in 50 μL decoloring solution [15 mM K_3_Fe(CN)_6_ and 50 mM NaS_2_O_3_] at RT for 5 min. The gel pieces were dried in 50% acetonitrile (ACN) at RT for 30 min and then dried in 100% ACN at RT for 30 min. Protein was digested using 0.4 μg sequencing grade trypsin at 37°C for 16 h. The digested peptides were extracted from the gel pieces using Extraction Solution (water solution containing 5% trifluoroacetic acid and 67% ACN) at 37°C for 30 min and then applied to the on-line liquid chromatography (LC)-collision-induced dissociation (CID)-MS-MS analysis.

### On-line LC-CID-MS-MS analysis

High-performance liquid chromatography was carried out to isolate trypsin-digested protein fragments on a 75 μm inner diameter 15 cm reverse-phase capillary column, 3.0 μm, 120 Å, C18 (ChromXP Eksigent) using a gradient from 5 to 35% buffer B (98% ACN, 0.1% formic acid) over a period of 90 min. Mass spectrometry was performed on a TripleTOF5600 system (AB SCIEX) using electrospray ionization. The optimum conditions: ion spray voltage, 2.5 kV; curtain gas, 30 psi; nebulizing gas (GS1), 5 psi; interface heater temperature, 150°C. For information dependent acquisition, survey scans were acquired in 250 ms. At most, 35 product ion scans were collected exceeding a threshold of 150 counts/s (2^+^~5^+^ charges). Total cycle time was set to 2.5 s. For CID, a rolling collision energy setting was applied to precursor ions. Proteins were identified using the MASCOT V2.3 search engine (Matrix Science Ltd., London, UK.). Search parameters were as follows: two missed cleavage sites; fixed modifications of Carbamidomethyl (C); partial modifications of Acetyl (Protein N-term), Deamidated (NQ), Dioxidation (W), Oxidation (M); precursor ion tolerance is ± 30 ppm, fragment ion tolerance is ± 0.15 Da.

### Statistical analysis

All results are expressed as mean ± standard error of the mean (SEM). Statistical analyses were performed using GraphPad Prism statistical software 7.0 (Graphpad Software, La Jolla, California, USA). The Shapiro–Wilk normality test or the Kolmogorov–Smirnov normality test was used. Data were analyzed using unpaired *t*-test, Mann–Whitney test, one-way ANOVA followed by Tukey's multiple comparisons test or Kruskal–Wallis test followed by Dunn's multiple comparisons test wherever applicable. *P* < 0.05 was considered statistically significant.

## Results

### Hypoxia (10% oxygen level) significantly increased migration of vascular endothelial cells

**Figure 1:**
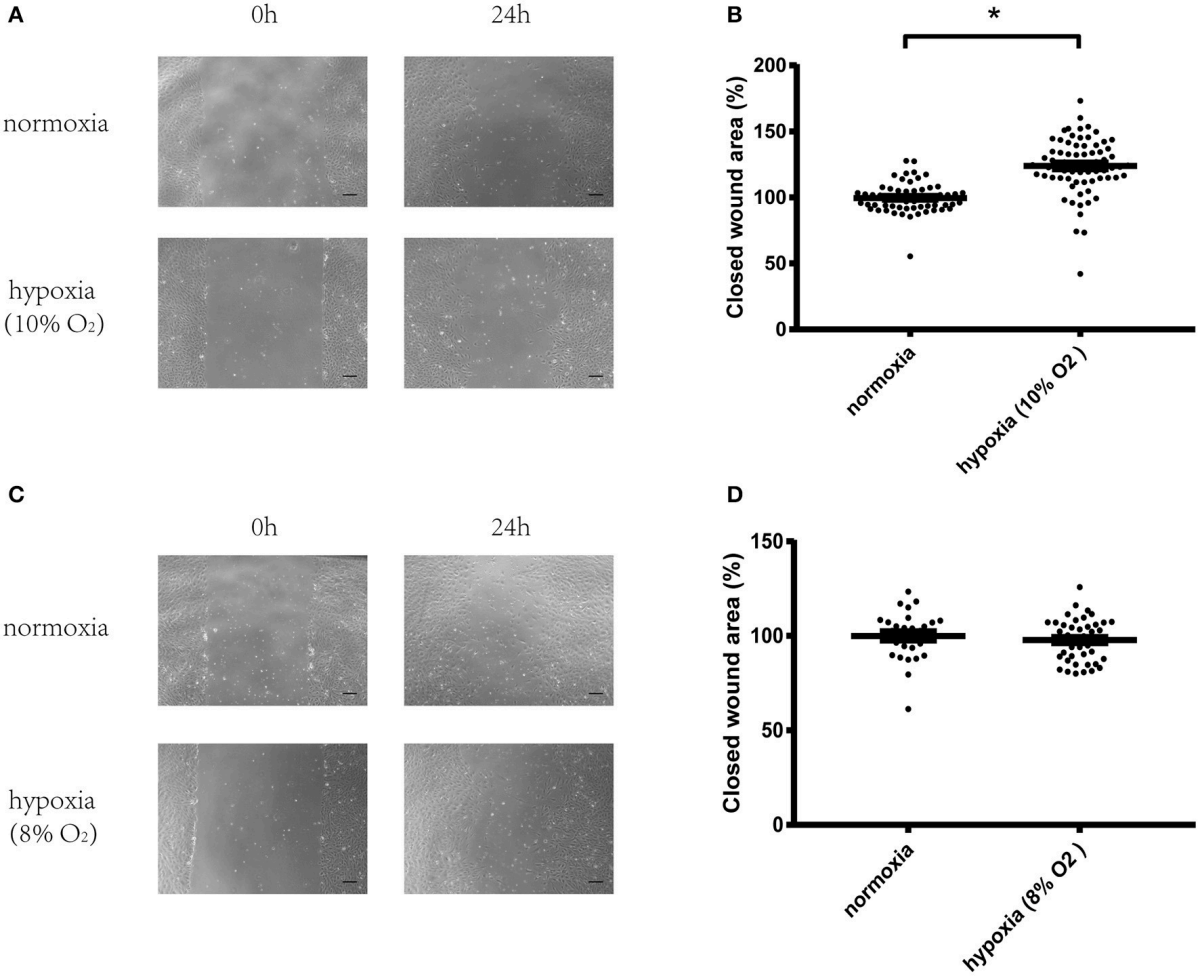
Hypoxia (10% oxygen level) could significantly increase migration of vascular endothelial cells. **(A)** Micrographs of wound healing experiments with vascular endothelial cells cultured in normoxia or hypoxia (10% oxygen level). **(B)** Hypoxia (10% oxygen level) could significantly increase the migration of vascular endothelial cells compared with cells cultured in normoxia. **(C)** Micrographs of wound healing experiments with vascular endothelial cells cultured in normoxia or hypoxia (8% oxygen level). **(D)** Hypoxia (8% oxygen level) exerted no effect on the migration of vascular endothelial cells. A total of 8–12 visual fields were chosen at random in one culture dish. The value of each wound area was converted to a standard value shown as one data point through division by a fixed value which renders the average value of the control group to be 100. The data are described as mean ± SEM. Bar = 200 μm. ^*^P < 0.05.

After starving overnight, HUVECs were scraped using a pipette tip for the wound healing assay. Then, the cells were cultured in normoxic or hypoxic conditions with 8 or 10% oxygen levels. Results showed that hypoxia (10% oxygen level) significantly increased cell migration compared with cells cultured in normoxia (Figures 1A,B). However, more severe hypoxia (8% oxygen level) exerted no effect on cell migration (Figures 1C,D). Therefore, 10% oxygen level was chosen for the following experiments.

### RNA interference of CSE significantly increased migration of vascular endothelial cells in both normoxic and hypoxic conditions

**Figure 2:**
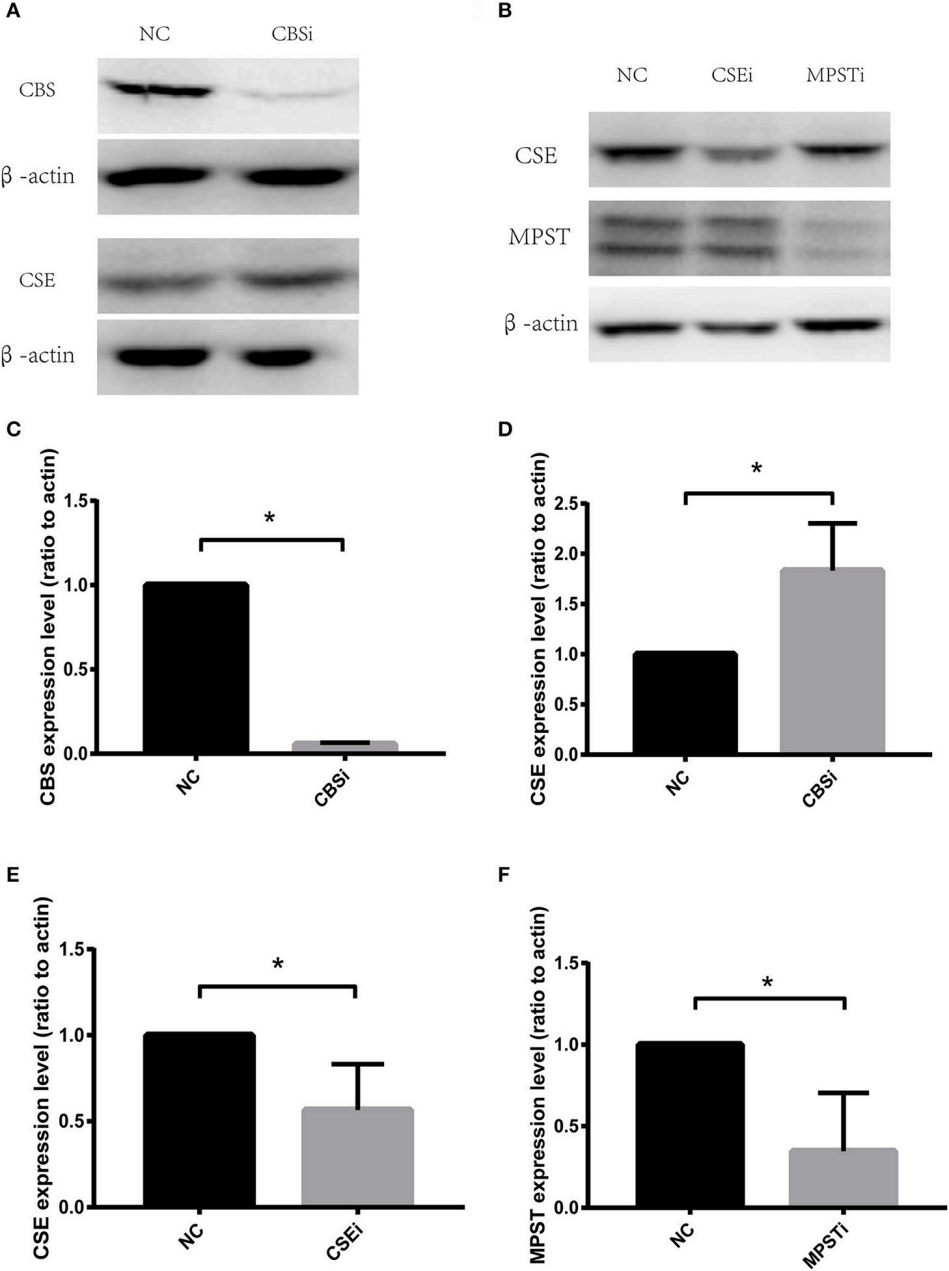
RNA interference of cystathionine γ-lyase (CSE), cystathionine β-synthase (CBS), and 3-mercaptopyruvate sulfurtransferase (MPST). **(A)** Representative bands of western blot analysis showing the effect of CBS RNA interference on CBS and CSE protein levels. **(B)** Representative bands of western blot analysis showing the effect of CSE or MPST RNA interference on CSE and MPST protein levels. **(C)** Successful knockdown of CBS using RNA interference. **(D)** Knocking down CBS has no significant effect on CSE protein levels. **(E)** Successful knockdown of CSE using RNA interference. **(F)** Successful knockdown of MPST using RNA interference. The histograms show the intensity ratio of the target protein to β-actin. NC, transfecting negative control; CSEi, transfecting CSE siRNA; CBSi, transfecting CBS siRNA; MPSTi transfecting MPST siRNA. The data are described as mean ± SEM, *n* = 4. ^*^P < 0.05.

**Figure 3:**
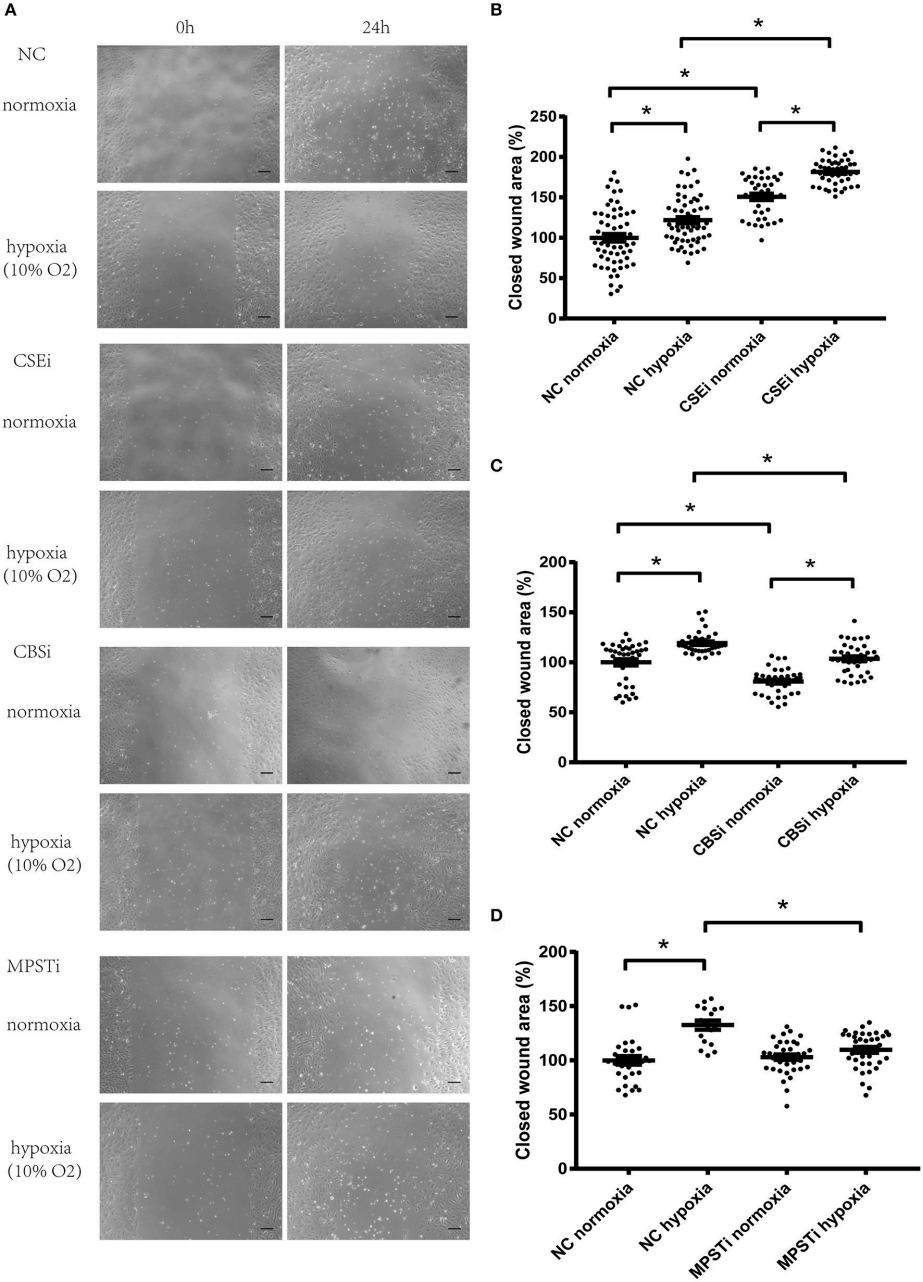
Role of CSE, CBS, and MPST in the migration of vascular endothelial cells in normoxia or in hypoxia. **(A)** Micrographs of wound healing experiments with vascular endothelial cells cultured in normoxia or hypoxia (10% oxygen level). **(B)** RNA interference of CSE significantly increased the migration of vascular endothelial cells in both normoxic and hypoxic conditions, and hypoxia-induced increase in migration was not mediated by CSE. **(C)** RNA interference of CBS significantly inhibited the migration of vascular endothelial cells in both normoxic and hypoxic conditions, and hypoxia-induced increase in migration was not mediated by CBS. **(D)** RNA interference of MPST significantly decreased the migration of vascular endothelial cells in hypoxic conditions, but had no effect in normoxic conditions. Moreover, hypoxia-induced increase in migration was mediated by MPST. NC, transfecting negative control; CSEi, transfecting CSE siRNA; CBSi, transfecting CBS siRNA; MPSTi transfecting MPST siRNA. A total of 8–12 visual fields were chosen at random in one culture dish. The value of each wound area was converted to a standard value shown as one data point through division by a fixed value which renders the average value of the control group to be 100. The data are described as mean ± SEM, ^*^P < 0.05.

CSE protein levels were significantly decreased using RNA interference (Figures 2B,E). HUVECs transfected with negative control siRNA (NC) or CSE siRNA for 36 h were starved overnight, and then used in a wound healing assay. Results showed that CSE knockdown by RNAi (CSEi group) significantly increased the migration of endothelial cells compared with the NC group in both normoxia (NC normoxia vs. CSEi normoxia) and hypoxia (NC hypoxia vs. CSEi hypoxia; Figures 3A,B). These results suggest that the endogenous hydrogen sulfide produced by CSE might probably play an inhibitory role in the migration of vascular endothelial cells.

### RNA interference of CBS significantly decreased migration of vascular endothelial cells in both normoxic and hypoxic conditions

CBS protein levels were significantly decreased using RNA interference (Figures 2A,C). HUVECs transfected with negative control siRNA (NC) or CBS siRNA for 36 h were starved overnight, and then used in a wound healing assay. Results showed that CBS knockdown by RNAi (CBSi group) significantly decreased the migration of endothelial cells compared with the NC group in both normoxia (NC normoxia vs. CBSi normoxia) and hypoxia (NC hypoxia vs. CBSi hypoxia; Figures 3A,C). These results suggest that the endogenous hydrogen sulfide produced by CBS might probably play a stimulatory role in the migration of vascular endothelial cells.

### RNA interference of MPST significantly decreased migration of vascular endothelial cells in hypoxic conditions, but has no effect in normoxic conditions

MPST protein levels were significantly decreased using RNA interference (Figures 2B,F). HUVECs transfected with negative control siRNA (NC) or MPST siRNA for 36 h were starved overnight, and then used in a wound healing assay. Results showed that MPST knockdown by RNAi (MPSTi group) significantly decreased the migration of endothelial cells compared with the NC group in hypoxia (NC hypoxia vs. MPSTi hypoxia; Figures 3A,D). However, there was no difference between the NC group and the MPSTi group in normoxia (NC normoxia vs. MPSTi normoxia; Figures 3A,D). These results suggest that the endogenous hydrogen sulfide produced by MPST might probably turn into effect in promoting the migration of vascular endothelial cells once oxygen levels have decreased.

### Hypoxia-induced increase in migration of vascular endothelial cells was mediated by MPST, not CSE or CBS

HUVECs in hypoxia migrated faster than those in normoxia after transfection with NC siRNA (NC normoxia vs. NC hypoxia; Figure 3). Knocking down MPST using siRNA could abrogate the hypoxia-induced increase in the migration of HUVECs (Figures 3A,D). However, after knockdown of CSE or CBS, the hypoxia-induced increase in endothelial cell migration persisted (Figures 3A–C). These results suggest that the endogenous hydrogen sulfide produced by MPST might probably mediate the hypoxia-induced increase in the migration of vascular endothelial cells, while hydrogen sulfide produced by CSE or CBS has no significant effect.

### MPST protein levels were significantly elevated by hypoxia in a bi-phasic manner

**Figure 4:**
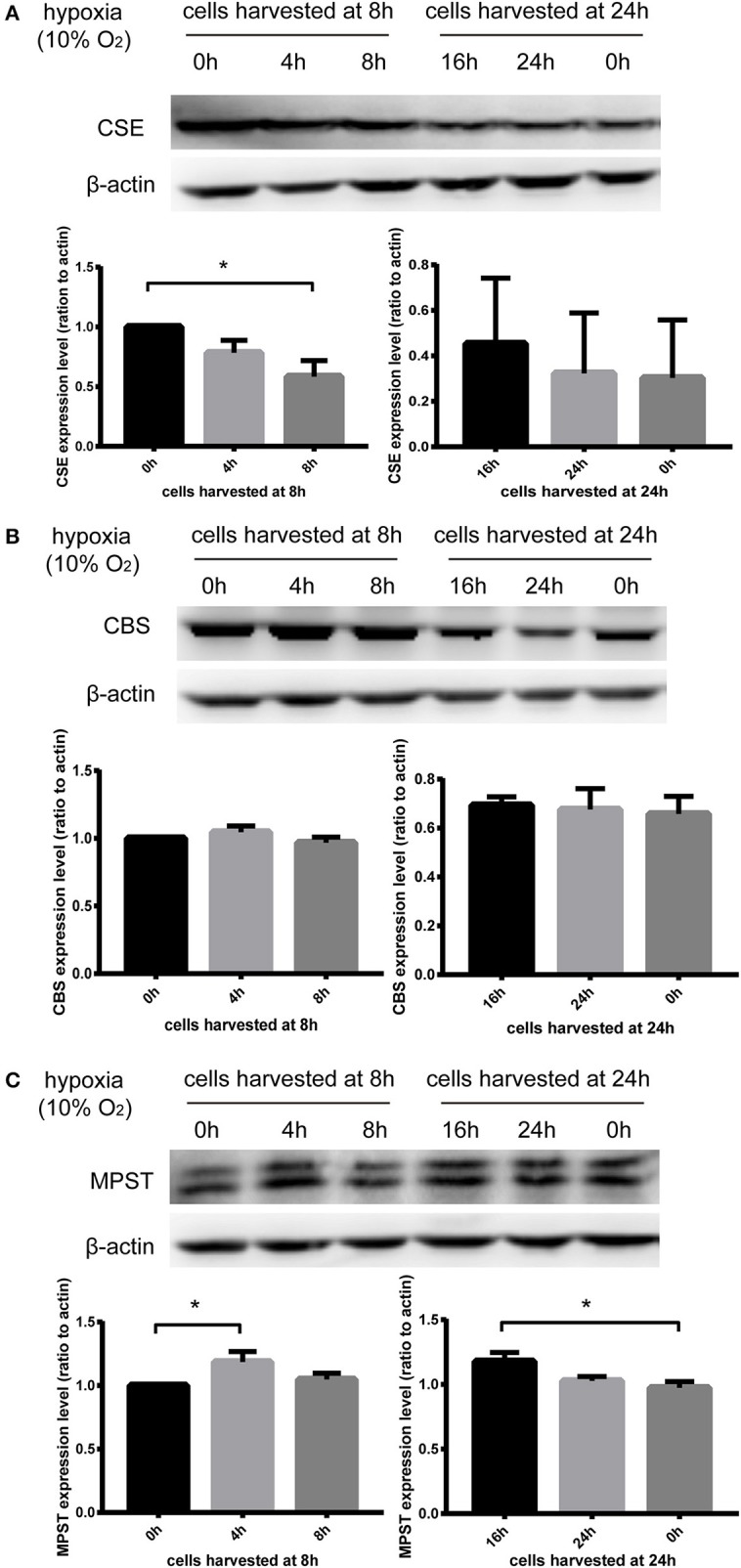
Effect of hypoxia (10% oxygen level) on CSE, CBS, and MPST protein levels. Vascular endothelial cells starved overnight were scratched. In order to exclude the impact of starvation time on the expression of CSE, CBS, and MPST, cells were placed in the incubator for hypoxia treatment at different time points and harvested at the same time (8 or 24 h). **(A)** Hypoxia could significantly decrease the protein levels of CSE at 8 h. *n* = 5. **(B)** Hypoxia had no effect on the protein levels of CBS. *n* = 6. **(C)** Hypoxia significantly increased the protein levels of MPST at 4 and 16 h. *n* = 4. The histograms show the intensity ratio of the target protein to β-actin. The data are described as mean ± SEM, ^*^P < 0.05.

In order to exclude the impact of starvation time on the expression of CSE, CBS, and MPST, cell dishes were placed in the incubator for hypoxia treatment at different time points and harvested at the same time (8 or 24 h). Therefore, the cells were cultured in hypoxia for the times indicated in Figure 4. MPST protein levels were significantly increased after culturing in hypoxia for 4 or 16 h (Figure 4C). In addition, hypoxia could significantly decrease CSE protein levels at 8 h (Figure 4A), but had no effect on CBS protein levels (Figure 4B). These results suggest that the three endogenous hydrogen sulfide producing enzymes CSE, CBS, and MPST respond differently to hypoxia and the bi-phasic elevation of MPST implies a more complicated mechanism in the regulation of its protein level.

### MPST mRNA levels were significantly increased by hypoxia later than the first stage elevation of its protein levels

**Figure 5:**
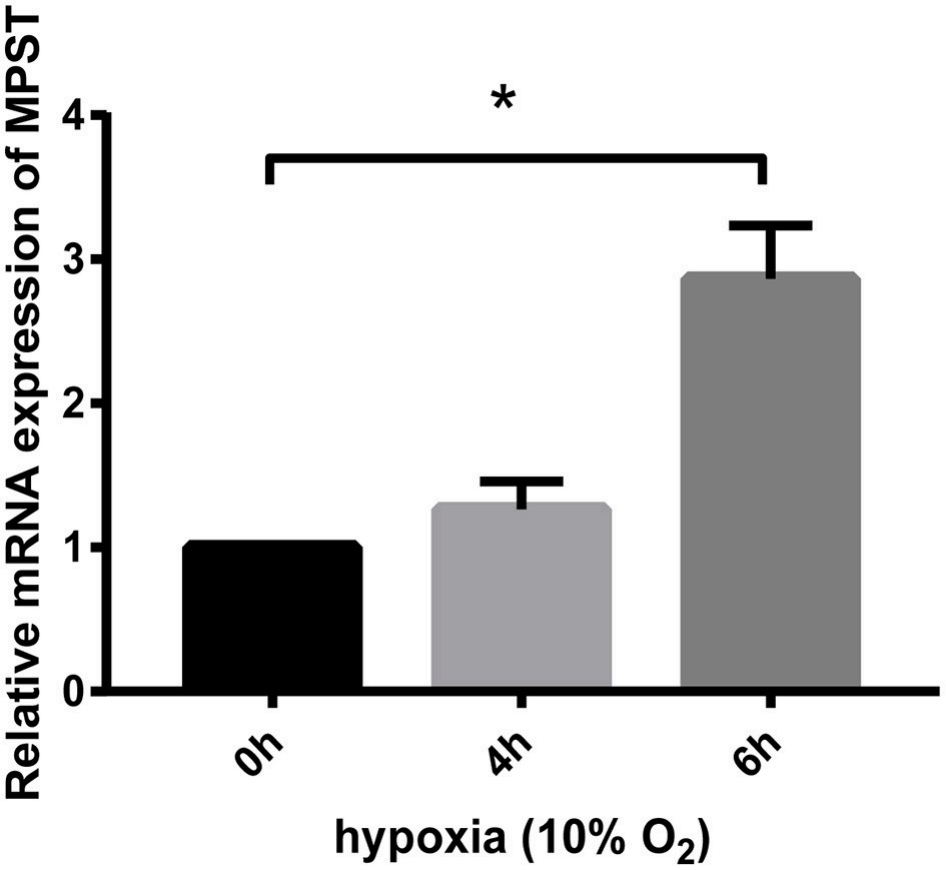
Effect of hypoxia (10% oxygen level) on the mRNA levels of MPST. GAPDH was used as a reference for normalization and the relative expression of mRNA was calculated according to the ΔΔCt method. *n* = 13 at 4 h; *n* = 15 at 6 h. The data are described as mean ± SEM, ^*^P < 0.05.

HUVECs starved overnight were treated using Trizol reagent for further real-time PCR analyses. Results showed that MPST mRNA levels were not increased at 4 h in hypoxia when MPST protein levels had already been elevated (Figure 5). These results suggest that the first-stage elevation of MPST protein levels was probably induced through a post-transcriptional mechanism. However, MPST mRNA levels were increased at 6 h in hypoxia (Figure 4), which suggests that the later-stage elevation of MPST protein levels might probably be induced at a transcriptional level.

### Hypoxia significantly decreased binding proteins of the 5′UTR of MPST mRNA

**Figure 6:**
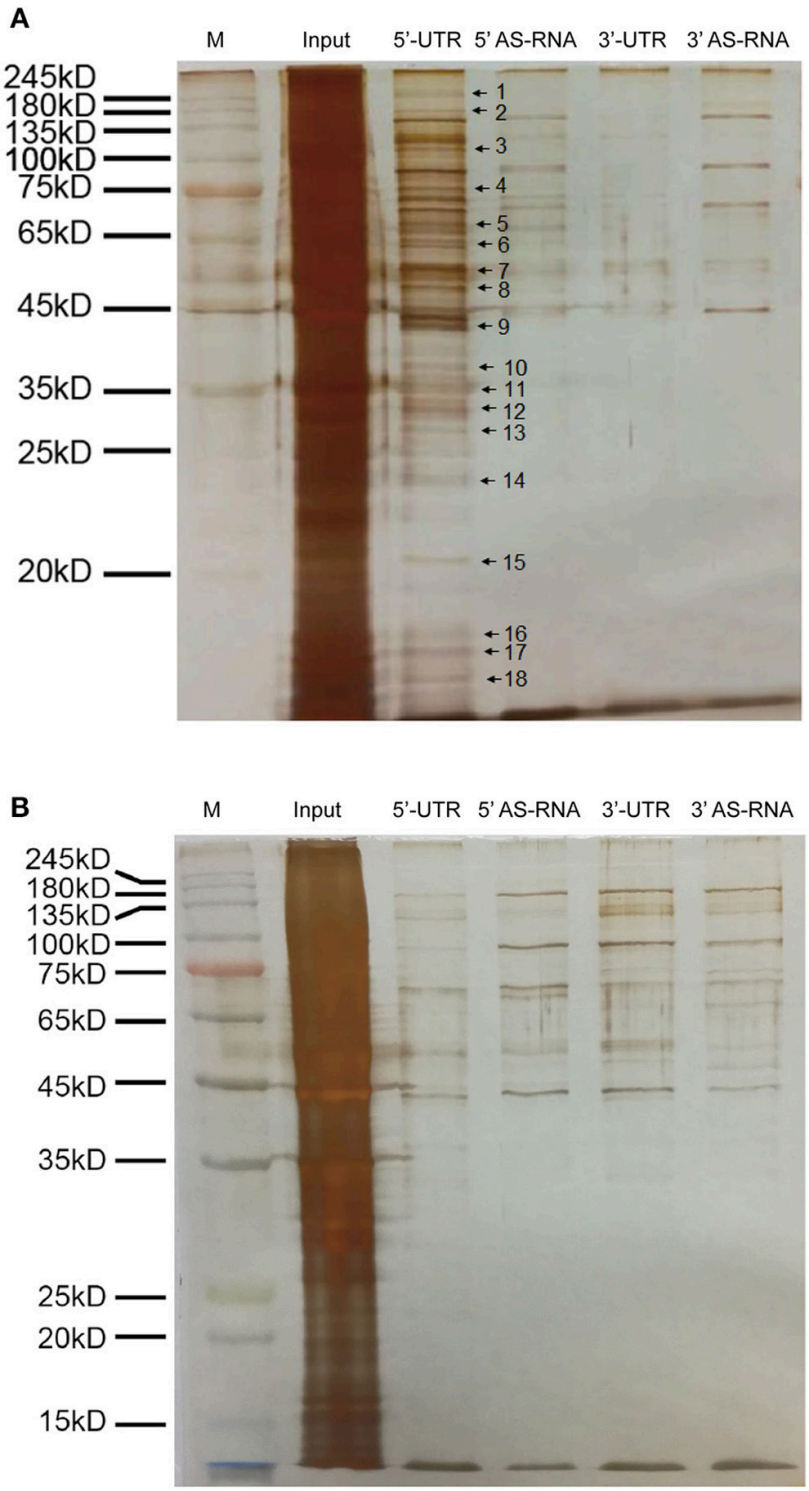
Hypoxia significantly decreased the binding proteins of the 5′UTR of MPST mRNA. **(A)** Micrographs of a silver stained SDS-PAGE gel. The UTR of MPST mRNA was incubated with vascular endothelial cells cultured in normoxia. **(B)** Micrographs of a silver stained SDS-PAGE gel. The UTR of MPST mRNA was incubated with vascular endothelial cells cultured in hypoxia. Input, cell lysates. 5′UTR, RNA pull-down experiments using the 5′UTR of MPST mRNA. 5′AS-RNA, RNA pull-down experiments using the antisense sequence of the 5′UTR of MPST mRNA. 3′UTR, RNA pull-down experiments using the 3′UTR of MPST mRNA. 3′AS-RNA, RNA pull-down experiments using the antisense sequence of the 3′UTR of MPST mRNA. Protein bands indicated by the arrows were chosen for LC-MS/MS analyses.

To investigate the post-transcriptional regulation of MPST mRNA by hypoxia, the 5′UTR and 3′UTR of MPST mRNA with biotin tags were prepared. RNA pull-down experiments were performed by incubating biotin-tagged mRNA with lysates of HUVECs after culturing in normoxic or hypoxic conditions. The antisense sequence of the UTR region was used as a negative control. The mixture was then incubated with streptavidin-coated beads to obtain biotin-tagged RNA with binding proteins on it. SDS-PAGE with silver staining showed that many proteins bound with the 5′UTR, rather than the 3′UTR of MPST mRNA in normoxic conditions compared with their antisense RNA groups (Figure 6A). However, those binding proteins were significantly decreased after cells were cultured in hypoxia for 4 h (Figure 6B).

### Candidate RNA binding proteins of MPST mRNA

In order to identify the binding proteins of MPST mRNA which were significantly decreased at the protein level or dissociated from MPST mRNA, the protein bands indicated with arrows and numbers in Figure 6A were chosen for in-gel digestion using trypsin and then analyzed through LC-MS/MS. All proteins proposed by the mass spectrometry data and Mascot searching engine were ranked by score and are presented in the Supplementary Tables in the form of Excel files (candidates of bands no. 1 and 2 are shown in Supplementary Table 1; band no. 3, Supplementary Table 2; band no. 4, Supplementary Table 3; band no. 5, Supplementary Table 4; band no. 6, Supplementary Table 5; bands No. 7 and 8, Supplementary Table 6; band no. 9, Supplementary Table 7; band no. 10, Supplementary Table 8; bands no. 11, 12, and 13, Supplementary Table 9; band no. 14, Supplementary Table 10; band no. 15, Supplementary Table 11; bands No. 16, 17, and 18, Supplementary Table 12). Score and mass weight will be considered simultaneously in choosing the candidate RNA binding proteins of MPST mRNA, and whether hypoxia decreased the protein levels of candidates or inhibited the association of candidates with 5′UTR mRNA will be further investigated.

## Discussion

It has been previously reported that hydrogen sulfide has a significant promoting effect on the process of angiogenesis both *in Vitro* and *in Vivo* (Cai et al., 2007; Papapetropoulos et al., 2009). In addition, there is also accumulating evidence concerning the proangiogenic role of H_2_S in ischemic disease models (Wang et al., 2010; Bir et al., 2012; Jang et al., 2014). Signaling pathways which mediate the effects of H_2_S in angiogenesis have been investigated. It has been reported by Cai et al. that the PI3K/Akt pathway was involved in its proangiogenic effect, while VEGF elevation was not observed in vascular endothelial cells after H_2_S treatment (Cai et al., 2007). However, VEGF expression levels in skeletal muscle cells in the ischemic hind limb were significantly increased by H_2_S, indicating that the proangiogenic effect of H_2_S might involve cell-cell interactions between vascular endothelial cells and skeletal muscle cells (Wang et al., 2010). In addition, other proangiogenic factors are also involved. It was reported that VEGF, Ang-1 and Ang-2 expression levels were increased by H_2_S in astrocytes. In accordance with that, VEGF and Ang-1 in the ischemic brain were also elevated by H_2_S. Moreover, ERK and HIF-1 (Jang et al., 2014), the K_ATP_ channel and p38 (Papapetropoulos et al., 2009), and other gaseous signaling molecules including nitric oxide and carbon monoxide (Coletta et al., 2012), might also be involved in the proangiogenic effect of H_2_S.

Although, numerous signaling molecules involved in the effects of H_2_S have been reported, one of the most challenging questions in this field is the identification of “receptors,” namely the direct target of H_2_S. We reported previously that H_2_S could directly increase the activity of recombinant VEGFR2 in a cell-free system and found that there was a new disulfide bond C1045-C1024 in the kinase domain of VEGFR2 which could be broken by H_2_S, suggesting that VEGFR2 might be a “receptor” for H_2_S (Tao et al., 2013).

There are three endogenous hydrogen sulfide producing systems in mammalian cells, CSE/H_2_S, CBS/H_2_S and MPST/H_2_S. We have previously found that CSE and CBS were located differently in vascular endothelial cells (Tao et al., 2013). CSE appeared to be located near the cell membrane, while CBS was more evenly distributed in the cytoplasm. Therefore, it was hypothesized that H_2_S produced from these enzymes could not reach target proteins far from the enzyme, since H_2_S could be easily oxidized (Cuevasanta et al., 2017) and the concentration of H_2_S would be too low to have any effect. In the current paper, it was found that CSE, CBS and MPST were playing different roles in the migration of vascular endothelial cells. As shown in Figures 3A,B, RNA interference of CSE produced a significant promoting effect in cell migration both in normoxia and in hypoxia, suggesting that CSE/H_2_S was playing an inhibitory role in vascular cell migration. As shown in Figures 3A,C, RNA interference of CBS produced a significant inhibitory effect in cell migration both in normoxia and in hypoxia, suggesting that CBS/H_2_S was probably playing a promoting role in vascular cell migration. As shown in Figures 3A,D, RNA interference of MPST showed no effect on cell migration in normoxia, but had an inhibitory effect on cell migration in hypoxia, suggesting that MPST/H_2_S was playing a promoting role in vascular cell migration only in hypoxic conditions. These findings indicate for the first time that CSE, CBS and MPST play different roles in vascular cell migration, although they all produced H_2_S. It has been reported that the distinctive expression pattern of CSE and CBS is closely related to phenotype in human mesenchymal stromal cells. CSE up-regulation was restricted to those cells undergoing mineralization, while CBS, not CSE expression levels were significantly increased during chondrogenesis (Gambari et al., 2017). In addition, Cao et al. reported that butyrate-inhibited cell viability could be reversed by CBS blockade, while CSE blockade had no such effect (Cao et al., 2010). These findings suggest that CSE and CBS have different roles in mediating cell function, which is in accordance with our results.

In contrast with CSE and CBS, MPST localizes in mitochondria as well as in the cytosol (Nagahara et al., 1998; Shibuya et al., 2009b; Tanizawa, 2011). Vascular endothelial cells are sensitive to hypoxia and their mitochondria are involved in the response to hypoxia by initiating the angiogenic process (Caja and Enriquez, 2017). In addition, it has been reported that mitochondrial reactive oxygen species (ROS) production is involved in angiogenesis and the adaptive responses of endothelial cells to hypoxia and oxidative stress (Amanso and Griendling, 2012). Therefore, we speculated that H_2_S produced from MPST in mitochondria could decrease ROS levels that mediate hypoxia-induced endothelial cell migration. However, why MPST/H_2_S, but not CSE/H_2_S or CBS/H_2_S, mediated hypoxia-induced vascular endothelial cell migration and whether there are different specific direct target proteins, or “receptors,” for different H_2_S producing systems, will need to be further investigated.

It was found that the increase in MPST protein levels appeared earlier than the increase in MPST mRNA levels, suggesting that MPST might also be regulated at a post-transcriptional level. RNA pull-down experiments showed that there might be multiple RNA binding proteins on the 5′UTR of MPST mRNA, rather than the 3′UTR. Moreover, these binding proteins “disappeared” when vascular endothelial cells were cultured in hypoxia. Therefore, we speculate that those binding proteins might have a role in regulating MPST mRNA stability or translation and they might be regulated by hypoxia. Among those candidate binding proteins listed in the Supplementary Tables, some have been reported previously to be RNA binding proteins, such as nucleolin (Fahling et al., 2005; Jiang et al., 2014; Saha et al., 2016) and Annexin A2 (Hollas et al., 2006). However, their precise role in regulating MPST mRNA will need to be further investigated.

Figures 2A,D show that after CBS RNA interference, CSE protein level significantly increased. It has been reported that when CSE was knocked down in cardiomyocytes, the expression of CBS and MPST significantly increased in compensation (Li et al., 2016), which is in accordance with our results.

In the current study, it was found for the first time that CSE, CBS, and MPST played different roles in the migration of vascular endothelial cells in normoxia and hypoxia. In addition, MPST, not CSE or CBS mediated hypoxia induced cell migration, probably through a post-transcriptional mechanism.

## Author contributions

BT and YZ conceived and designed the experiment. BT, RW, and CS performed the experiments. BT analyzed the data. BT and YZ wrote the paper. All authors have contributed to the paper and approve the submission of the manuscript.

### Conflict of interest statement

The authors declare that the research was conducted in the absence of any commercial or financial relationships that could be construed as a potential conflict of interest.
